# For Gene Activation, Location Matters

**DOI:** 10.1371/journal.pbio.0020381

**Published:** 2004-09-28

Multicellular organisms contain a complete set of genes in nearly all of their cells, each cell harboring the potential to make nearly any protein in their genome. The same holds true for a single-celled bacterium or yeast. Yet a cell activates only a fraction of its genes at any given time, calling on a number of different mechanisms to activate the right genes at the right time. To metabolize sugar, for example, a cell needs to synthesize proteins involved in sugar metabolism, not protein repair, and vice versa. In a new study, Jason Brickner and Peter Walter report a mechanism for gene activation that depends on shuttling DNA to a particular location within the nucleus.

In organisms whose cells have nuclei (eukaryotes), genomes lie within the nucleus (called the nucleoplasm) but also interact with the inner nuclear membrane. Transcription factors activate gene expression by binding to a promoter sequence in the gene's DNA. The physical structure of DNA—which is packaged with proteins into chromatin—affects gene expression by controlling access to DNA. Where chromatin exists in the nucleus also influences gene expression. Heterochromatin—stretches of highly condensed chromatin—typically lines the nuclear periphery, and genes bundled into heterochromatin are typically silent. Active transcription generally occurs in the less condensed euchromatic regions. But since euchromatic regions are also silenced when they associate with heterochromatin along the membrane, it is thought that delivering chromatin to the nuclear periphery regulates transcriptional repression. Brickner and Walter, however, found evidence of the opposite effect—recruiting genes to the nuclear periphery can promote their activation—suggesting that nuclear membrane recruitment plays a much broader role than previously suspected in gene regulation.

**Figure:**
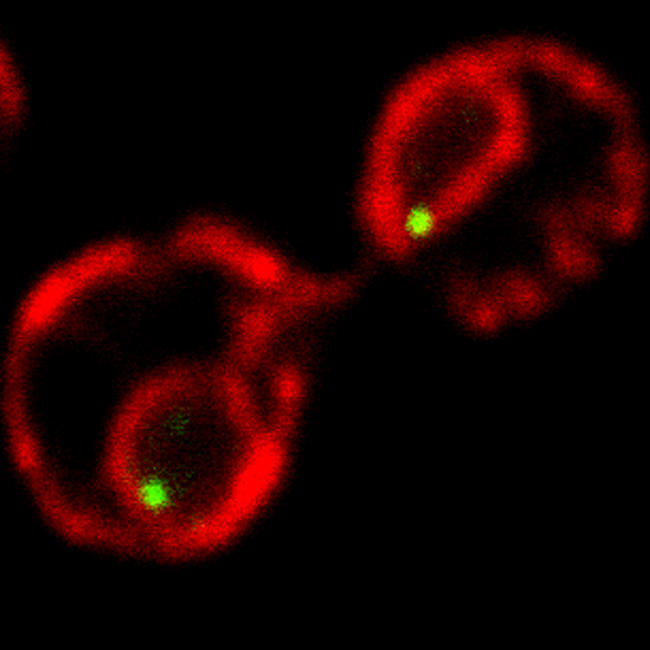
The INO1 gene (green) is recruited to the nuclear membrane (red) upon activation

To explore the consequences of chromatin location, the authors focused on a yeast gene called *INO1,* which encodes inositol 1-phosphate synthase, an enzyme involved in phospholipid (fat) biosynthesis. *INO1* is also a target gene of the “unfolded protein response,” which is triggered when unfolded proteins accumulate in the endoplasmic reticulum, a subcellular organelle where secreted proteins are folded. The *INO1* gene contains a regulatory element (called UASINO) within its promoter region that responds to inositol availability. Genes under the control of this element are transcriptionally repressed by a repressor, Opi1, and activated by two transcription factors, Ino2 and Ino4. The presence of unfolded proteins sets off a chain of events to relieve Opi1 repression and allow activation of *INO1*.

Through a series of genetic and biochemical studies, Bricker and Walter show that Ino2 and Ino4 are always bound to the *INO1* promoter. Opi1 associates with the chromatin, restricting the *INO1* locus to the nucleoplasm and repressing transcription. Induction of the unfolded protein response bumps Opi1 off the chromatin and, with Opi1 out of the way, *INO1* travels to the membrane and transcription proceeds. Crucially, the authors show that artificial recruitment of *INO1* to the nuclear membrane can be enough to activate the gene. There are several mechanistic aspects of this model to figure out still, but Brickner and Walter argue that for *INO1,* gene recruitment to the nuclear membrane promotes its activation. In light of other recent work, this phenomenon may be emerging as a more general mechanism for regulating eukaryotic gene expression.

